# Elderly Care Practitioners’ Perceptions of Moral Distress in the Work Development Discussions

**DOI:** 10.3390/healthcare11030291

**Published:** 2023-01-18

**Authors:** Elina Weiste, Maria Paavolainen, Nina Olin, Eveliina Korkiakangas, Eveliina Saari, Tiina Koivisto, Jaana Laitinen

**Affiliations:** Finnish Institute of Occupational Health, P.O. Box 40, 00032 Helsinki, Finland

**Keywords:** elderly care, ethics, emotions, qualitative research, healthcare professionals, interaction, moral distress, occupational health

## Abstract

Elderly care practitioners are at specific risk of experiencing prolonged moral distress, which is associated with occupational health-related problems, low job satisfaction, and staff turnover. So far, little attention has been paid to the moral concerns specific to elderly care, a field whose importance is constantly growing as the populations in Western countries age. By drawing on seven workshop conversations as data and interaction-oriented focus group research, conversation analysis and discursive psychology as methods, we aim to study the ways in which elderly care practitioners discuss moral distress in their work. We found that the moral distress experienced was related to three topics that arose when client work and teamwork contexts were discussed: the power to influence, equal treatment of people, and collaboration. The interaction in client work and teamwork contexts differed systematically. The discussion on client work was characterised by negotiations on the rights and wrongs of care work, whereas the teamwork discussion engendered emotional outbursts, a potential manifestation of work-related burnout. Hence, attempts to improve the work-related health of elderly care practitioners require time and space for sharing the emotional load, followed by reflection on what could be improved in the work and what institutional solutions could help in morally distressing situations.

## 1. Introduction

Ethics and moral problems are not only unavoidable but essential in the healthcare sector and in nursing. Healthcare professionals inevitably face choices that affect the wellbeing, rights, and lives of others, often the most vulnerable [1]. No matter how well healthcare institutions are funded, regulated or managed, there will always be uncertainty and disagreement over the selection, interpretation and application of values and principles. Values affect the way in which people define quality of life, good care and right and wrong conduct, and there are no neutral criteria for deciding which viewpoint is morally superior [2].

Ethical viewpoints are especially prominent in elderly care. Ageing reduces functional ability and increases the likelihood of chronic diseases and death, thus making patient autonomy and end-of-life issues common [3,4]. A recent study showed that elderly care practitioners experience moral distress (MD) more often than employees working in other healthcare sectors [5]. However, only little attention has been paid to the moral concerns faced in elderly care [6]. This is important, as ageing populations in Western countries are posing new ethical challenges to the elderly care sector. The number of nursing professionals is decreasing, creating staff shortages, while the need for care is increasing rapidly, e.g., [7]. In this article, we focus on elderly care practitioners’ perceptions of the challenging moral situations in their work.

Challenging moral situations can lead to MD. The term moral distress has been used to refer to situations in which a person is constrained from acting on what they think is right or in which they are uncertain of the right solution to the moral problem [8]. Andrew Jameton [9] introduced the term in the 1980s, and in recent years, interest in the phenomenon has been growing. Most of the previous studies have been qualitative interview studies trying to establish an understanding of the morally challenging situations in healthcare, i.e., [10,11,12]. These situations include disagreements over clinical decisions [13,14,15], the use of coercive methods and the lack of informed consent—especially in the case of individuals who may have cognitive impairment or dementia [16]. MD has also been linked to organisational factors such as lack of resources, insufficient staffing and a low degree of employee autonomy [11,17]; lack of support from co-workers and supervisors [16,18,19]. Understaffing, in particular, creates ethical challenges when nurses are forced to do things in a rush and compromise on the quality of care because of a lack of time [20].

Individual factors that cause MD include age, experience, competence and moral sensitivity. The lack of ethical competence—the set of skills and knowledge needed for recognising, analysing and reflecting on moral situations and the willingness to act according to one’s responsibilities [21,22]—may enhance MD. Existing research also recognises the critical role played by moral sensitivity. Moral sensitivity means one’s ability to recognise ethical perspectives and moral problems. Highly morally sensitive persons are more likely to be emotionally affected by moral shortcomings, which can result in more intensely experienced MD [23,24]. The correlation between MD, age and work experience is not well established. Some studies have suggested that older nurses’ distress is higher in intensity, i.e., [25], whereas other studies have found positive associations between MD and younger and novice nurses [26,27]. 

Experiences of MD often have negative consequences for nursing practitioners. MD may invoke negative emotions, including helplessness, guilt, frustration, anger and a feeling of being emotionally drained [14,16,28]. When incessant, these types of negative emotional experiences may be a possible sign of occupational burnout [29,30]. If MD experiences remain unaddressed in the workplace and become prolonged, they may lead to low job satisfaction and staff turnover [31,32], reduced work ability [5] and health conditions such as insomnia, anxiety depression and burnout [17,33]. Although MD is linked to occupational burnout, these two phenomena can also be separated. Burnout is a prolonged response to chronic work stress and can result from numerous factors such as heavy workload, low predictability and lack of work community. Ethical aspects may play a part in burnout but are not a prerequisite for it. [34] In contrast, MD is always rooted in constraint on one’s moral agency [35].

The ways in which nurses cope with MD vary. For instance, in the context of psychiatric nursing, Deady and McCarthy [15] found that a common strategy was “immunising” oneself to the moral conflict by avoiding the source of the conflict, denying it, refusing to work with a particular co-worker or leaving the post or the nursing profession altogether. MD can also lead to moral numbness and disengagement, which refers to a psychological process in which an individual ”justifies one’s unmoral actions by altering one’s moral perception of those actions” [36] (p. 15). However, not all consequences of MD are negative. Some research has pointed out that the experience of MD can also make nurses more sensitive to moral issues and motivate them to act ethically [24,37]. MD has been described as a “troubled call of conscience” [37] (p. 16) that reveals our deepest values and warns us when these values are being compromised. This is important, as it is our deepest values that motivate us to exercise our moral agency [24].

As unaddressed experiences of MD may have dramatically negative consequences for nursing practitioners, researchers have proposed a range of practices to alleviate MD. These include ensuring adequate resources [16,38], improving interprofessional ethical dialogue [2,15], see also [19], developing ethical principles and guidelines, making time for discussing moral problems with co-workers and increasing ethical competence among nursing professionals [2,33,36]. It is vital that we point out here that MD and the lack of ethical competence is not the problem of the individual nurse. For instance, Klotz and colleagues [21] emphasise the responsibilities of nursing schools for developing appropriate curriculum designs that support ethical competence. They argue that it is imperative that nursing students are taught how to apply ethical knowledge and decision-making in their future profession, but that there should also be a greater focus on teaching coping strategies that support students’ moral resilience, i.e., their abilities to endure moral uncertainties and challenges, and abilities to recover from them. The writers advocate establishing professional nursing associations and making them responsible for creating evidence-based, comprehensive ethical competency standards for nursing education programmes [21].

As noted above, MD among nursing practitioners has been studied from multiple perspectives, and its causes and consequences and strategies for its alleviation have been considered. Our work contributes methodologically and empirically to the discussion on MD among nursing practitioners in four important ways. First, it represents an in-depth qualitative investigation of the ways in which nursing practitioners themselves discuss MD in their work in joint development workshops. Second, by applying interaction-oriented focus group research, conversation analysis and discursive psychology, we can understand not only the contents that the elderly care practitioners relate to the concept of MD, but also the interactional dynamics that talking about the subject creates among them. In this way, we can focus on their joint construction of the meaning of MD in their work rather than their personal experiences and opinions. Third, this methodological combination gives us opportunities to analyse the topics that are associated with the normative viewpoints and emotional stances that the practitioners take towards MD, providing new information on the emotions related to MD. Fourth and finally, our work focuses specifically on nursing practitioners working in elderly care, who are known to experience MD more often than employees working in other healthcare sectors.

Our research questions are: (1) what do elderly care practitioners talk about when they talk about MD in their work, (2) how do they talk about it, and (3) are the different kinds of affective stances and normative orientations towards MD shared among elderly care practitioners?

## 2. Materials and Methods

### 2.1. Methodological Approach

To be able to link our analysis to both the content of the workshop members’ utterances and the patterns of interaction that they create [39,40,41], we used an appropriate combination of qualitative methods deriving from interaction-oriented focus group research [42,43], conversation analysis [44,45,46] and discursive psychology [47]. This combination of methodological approaches draws on the idea that there is an “inherent connection between the substantive content of what a person says and the interactive dynamics of how he or she says those things” [42] (p. 718). Thus, in addition to analysing the content of the workshop members’ talk, we are able to analyse how the participants “display how they align themselves toward other participants with whom they are interacting” [48] (p. 16).

In line with this approach, we are interested in the ways in which the participants negotiate the social norms and normativity of their work, and the affective expressions related to these negotiations. We understand normativity as “the judgments by which individuals designate some actions or outcomes as good, desirable, or permissible and others as bad, undesirable, or impermissible” [49] (p. 3). We do not see social norms as fixed observable categories, but as something that is constructed, challenged and reformulated in and through interaction [47]. Similarly, we see emotions and the construction of an emotional stance as a process that both shapes and is shaped by the interactional context [50]. Our interest in this article is the expressions of the normative and emotional stances that the participants take towards what they report, and the stances that the recipients take in their next turns of talk [51]. The idea is that the recipients may take a stance that matches (or does not) the teller’s stance toward the event, i.e., they may affiliate (or disaffiliate) with the teller [51]. We are also interested in the ways in which some views on MD mobilise mutually congruent assertions of consensus among workshop participants, whereas other views are received with explicit expressions of resistance and moral contempt or implicit expressions of indifference through, for example, silence [52]. The views that are preceded and followed by explanations and accounts are also of interest as they may demonstrate a participant’s need to justify their views in front of the other participants see, e.g., [53,54].

The micro-level analysis of the participants’ discussions on MD thus focuses on topics that are, on the one hand, associated with congruent affective stances and normative viewpoints, and on the other hand, involve subtle discrepancies between workshop participants.

### 2.2. Research Context and Materials

Our analysis was based on a dataset of seven audio-recorded and transcribed co-development workshop discussions. The workshops were held in one hospital district in Finland as part of the “Ensuring the availability of staff and the attractiveness of the sector in elderly care services” project, which is an implementation project of the National Programme on Ageing 2030, funded by the Finnish Ministry of Social Affairs and Health. The overall aim of the project was to develop an operational model for improving the wellbeing of employees in elderly care services and the appreciation and attractiveness of elderly care work.

The topic of the workshop series was “ethical work culture,” and the sessions aimed to create practices that support a culture change in elderly care. The workshops provided an opportunity to hear participants’ different perspectives on work, work-related wellbeing and the areas that they needed to develop. These discussions were used as a basis for designing experiments in the workplace [55,56]. The workshops applied the developmental dialogue method [57], the development map method [58] and principles of resource- and solution-focused work. The working methods aimed to support employee participation and empowerment.

In practice, each workshop process involved two meetings. The aim of the first workshop meeting was to identify the morally problematic situations at work and their effects at the employee and work community levels. In the second workshop, solutions and approaches to managing moral problems at work were identified. From these ideas, one to two development experiments were selected and jointly implemented at the workplace level. The workshops were face-to-face meetings in the organisations’ facilities. Each workshop process lasted three weeks in 2021.

This article focuses on only the first workshop because the first meeting was organised around a group discussion assignment on MD. In the assignment, the participants were asked to talk about their perceptions and experiences of morally distressing events in their work. The workshop participants were divided into small groups of four to five. The facilitators initiated the discussions on the assignments by giving instructions. The small groups discussed the assignment freely (without the facilitator being present) and made notes, after which each group shared the main points of their discussions with the whole group. Thus, the workshop discussions were relatively loosely structured, and the participants were able to choose how and how much to contribute to the discussions. The data of this article consist of seven such small-group discussions, each being approximately 50 min (350 min in total).

### 2.3. Research Participants

Two workshops were held in three different elderly care units. The units consisted of two service housing subunits and a geriatric ward. The service housing units were meant for the elderly population that need care services around the clock. Many of the clients had dementia or a progressive physical illness, restricting functional capacity in ways that meant that supported living at home was not an option. The geriatric ward mainly had patients in palliative or hospice care.

Each workshop contained 12–14 participants: one unit supervisor (*n* = 3) and 9–13 care workers (*n* = 35), totalling 38 participants, of whom 37 were female. The unit supervisors all had a nursing education and over 15 years of work experience in elderly care. They all participated in operative clinical work. The majority of the care workers were community nurses who had a three-shift work schedule. We have no information of their age and work experience. As the workshops aimed to improve organisational work practices, the participants were recruited from within the organisations, with no research-based inclusion or exclusion criteria. As each workshop involved participants from the same unit, the participants were familiar with each other. The facilitator was a well-experienced lecturer in nursing education. She gave the instructions but did not participate in the group discussions.

### 2.4. Research Ethics

The research was conducted in accordance with the Declaration of Helsinki, and permission to collect the data was obtained from the healthcare district and FIOH’s Ethics Committee (decision 18 December 2020). Informed, written consent was obtained from all participants before recording the workshops. They were also advised that they could withdraw their consent at any point during the data collection. All names and other details that could enable identification of the participants have been removed or altered in the text and data excerpts.

### 2.5. Analytic Process

During the analytical process, the first author (E.W.) first listened to the recordings several times and made notes on the segments during which the participants discussed their perceptions of “moral distress” in their work. Although the assignment given to them in the workshop focused specifically on this topic, some of the talk also revolved around other topics (such as the pre-Christmas party at the unit). For the purpose of this study, only the segments of interaction in which distressing work situations were the participants’ main topic were collected (*n* = 125). For a more detailed analysis of these segments, we used simplified conversation analytic transcription conventions [45] (see Appendix A), which necessitates focusing not only on talk, but also on nonverbal behaviour (e.g., voice quality) and turn transition points (e.g., overlapping talk and silences). After this, the first author (E.W.) analysed all the segments in a data-driven way, probing the topical categories and interactional patterns identified in a single data segment against every new segment of data. We also tested the intersubjective grasp of these categories with two analysts (E.W. and N.O.) independently coding pieces of data. This led to some further distributions into categories that we could reliably identify in our data.

## 3. Results

In the following, we present the results of our qualitative analysis in three sections, each of which focuses on one specific topic in the workshop participants’ discussion on MD that arose inductively from our analysis of the empirical data. The topics of the discussions on MD in the participants’ work were related to two contexts: client work and teamwork. In both contexts, the discussion revolved around three topics: the power to influence and make decisions, equal treatment of everyone, and collaboration (Table 1). Most of the discussion concerned the teamwork context, especially the elderly care practitioners’ ability to influence their work and the collaboration with their supervisors and colleagues. Collaboration with clients and their relatives also invoked a great deal of discussion.

Although, the discussions were similar topic-wise when they concerned client work or teamwork, they were different interactionally. When the participants talked about client work, the discussion was characterised by negotiations of social norms: they asked questions, provided justifications and pondered the rights and wrongs of care work. However, when they talked about teamwork, the interaction involved expressions of strong affective stances that were firmly affiliated with the other participants.

In what follows, we show how the members of our workshops discussed each of the three topics. In the subsections below, we show two data extracts on each topic to demonstrate the differences in the interactional underpinnings of the talk about distressing client work and teamwork. These data extracts are paradigmatic examples of the type of interactional segments found in the data. They were selected from the dataset as a whole to represent the findings in a clear and accessible way. The transcriptions in the data extracts were translated from Finnish by the authors.

### 3.1. Power to Influence

In our workshop data, the elderly care practitioners highlighted the importance of decision-making power. In the context of client work, this topic was connected to the clients’ self-determination. The practitioners pondered the balance between self-determination and nurture in cases in which the practitioners disagreed with the clients’ decisions about their own lives. They also discussed the relatives’ rights to make decisions concerning the lives of the clients, often over-ruling the clients’ own opinions. This topic mobilised negotiations of rights and wrongs, and the participants accounted for and justified their views. These accounts could be defined as “the social reasoning that people go through to make sense of their words and (perhaps) impose that sense on other people” [53] (p. 1). We are interested in how these accounts were used for reasoning the moral concerns in elderly care practitioners’ work.

The first data extract provides an example. Before the extract takes place, one of the participants has topicalised the visits of a demanding relative as a source of stress. In the first lines, another participant continues the topic and raises the question of whether the elderly care practitioners should listen to the client or the relatives when their views are in conflict.

Extract 1. (P = participant)

01 P1: When the relative says go and the client says

02    they don’t want to go, who do we listen to? If you don’t listen to the

03    relatives, there will be even bigger problems.

04 P2: If I think about this, I’d go with the client’s wish

05    because if they’ve said what

06    they want, then it’s their decision and I think

07    no one can over-rule it.

08    Quite often the decision is made according to the relative

09    if they don’t want to be taken anywhere, then they’re not.

10 P3: It’s similar to the eating thing, if a relative forbids

11    pancake and the client says they want to eat the pancake.

12 P2: So why can’t they eat it then?

13 P3: Because the relative has forbidden it

14    because they have diabetes (.) they’re not allowed to eat pancakes.

15 P2: So these are contradictions, but at the end of the day, it’s us that care for the client.

In lines 1–2, the participant (P1) describes a dilemmatic situation: the relative says that the client should go (she leaves the referent open and does not specify where the client should not go), although the client does not want to go. Then she formulates a question: whose view should the elderly care practitioners should listen to and support in practice? She favours listening to the relatives and justifies her view by stating that “it will cause more problems” for the practitioners if they listen to the client (line 3). P2 defends the opposite; she presents her view as her own thoughts and favours supporting the client’s decision (lines 4–6). She justifies her view by stating that another person cannot over-rule an individual’s decision (lines 6–7). She also argues that her view does not represent the general idea at the workplace: often, the decisions are made according to the relatives’ wishes (lines 8–9). At this point, another participant (P3) joins the discussion and agrees with P2′s account, justifying it by highlighting a specific eating situation at the workplace [59]: the client wanted to eat a pancake, but the relative objected (lines 10–11). P2 invites further justifications for the pancake example (line 12) and P3 responds by providing an explanation: the relative forbids the eating of pancakes because of the client’s diabetes (lines 13–14). At this point, P2 explicitly calls the discussed examples “conflicting issues” in their work and concludes that the client should be their priority (line 15). Thus, the client’s right to make decisions concerning their own life invoked normative accounts and moral pondering among the elderly care practitioners. The client’s decision-making power was considered a normative idea that the elderly care practitioners should promote in their work, but in some situations, it was experienced as burdensome.

When the elderly care practitioners talked about their decision-making power in their own work, they strongly agreed that they should be able to influence their work. Not being involved in decision-making was addressed with affective expressions. This is what happens in Extract 2, in which the workshop participants have discussed the recent changes in their workplace, including, for instance, COVID-19 restrictions and changes in the team structure.

Extract 2.

01 P1: I’ve said I have no problem if things are 

02  developed and there are positive changes, of course, things 

03  need to progress but firstly, the opinion of the

04  employees who do the work should be asked, for example 

05  by voting (0.2), we’ve suggested that we vote (.) 

06  our supervisor says ↓no, she decides that we do ↓it like this.

07  These changes may come every week, maybe three big changes, 

08  everything’s a mess (.) I told [name] straight when I came to work this 

09  weekend that it pisses me off to come to work.

10  I feel like I have no clue what I should do

11  when I come to work (.) then she’s like @we::::l that’s just the way it is@

12  ↑well OF COURSE as it’s NOT YOUR WORK that changes.

13  You just sit on your arse and tell us what to do

14  and we TOTALLY disa[gree.

15 P2: [It’s been TWO YEARS since she

16  worked with us on the ward [and, 

17 P3: [And only ONE

18  single shift in the summer.

At the beginning of the extract, P1 denies that she resists organisational changes per se. She claims to have no problem with development or positive changes at work, and strongly agrees that “things should progress” (lines 1–3). Next, she continues with the conjunction particle “but”, which expresses a divergent opinion [60] (p. 1098). She argues that in decision-making, ‘the employees who do the work should have a say, for instance, by voting (lines 3–5). She further justifies that she, among the other practitioners (referred to as “we”), had proposed voting at their workplace, but their supervisor refused. Instead, the supervisor appealed to her superior to make decisions (lines 5–6). This invoked a complaint about the supervisor, in which the other participants join in. P1 initiates the complaint by talking about the constant changes the supervisor makes at the workplace, causing everything to be “a mess” (lines 7–8). Next, she refers to a specific situation the previous weekend and with swear words describes her feelings when coming to work (lines 9–10). In addition, she makes an in-situ display of anger and indignation by using a high, loud and tense voice [61]. She also produces part of her description in a tone of voice (line 11, marked with @, see Appendix A) that mimics the voice of the supervisor when describing the situation, thus heightening the emotional tone of her description [62]. In line 15, P2 joins in the complaint, and by using the same high, loud and tense voice she affiliates with P1′s affective stance [51]. P3 also joins the discussion and completes P2′s sentence (lines 17–18). This type of co-completion demonstrates understanding [63] and a strong agreement between participants [64].

As demonstrated in Extract 1 and 2, the elderly care practitioners displayed an orientation to the ideal that people should have the right to influence decisions that concern their own lives. As this client’s right was threatened, it invoked accounts, justifications and disagreements when the practitioners pondered the social norms and morality of their work. When, however, the elderly care practitioners’ own rights to influence decisions concerning their own work was threatened, it invoked mutually shared expressions of emotions.

### 3.2. Equal Treatment of Everyone

Another topic that the workshop participants emphasised when talking about MD was equal treatment. Like the decision-making participation, it was presented as an ideal that should be realised in good quality healthcare. In the context of client work, violations of this ideal were expressed as distressing and addressed with normative accounts and moral pondering.

Extract 3 provides an example of this. At the beginning of the extract, one of the workshop participants initiates a new topic and designates the COVID-19 restrictions as a source of unequal treatment of clients in their ward.

Extract 3.

01 P1: I think the COVID restrictions have definitely been

02  one of the toughest things we’ve had here.

03 P2: They have, yes, I agree.

04 P1: It was somehow so hard that although we have clear- 

05  or instructions and guidelines for how to operate 

06  from a higher level, at least I think that they’re really difficult to –

07  we interpret them slightly differently (.) so are they equal?

08  And perhaps what bothered me the most – we discussed it

09  a lot in the ward when we had these [guidelines] that

10  the relatives of the hospice care patients were allowed to visit

11  around the clock but then palliative care patients’ relatives wer 

12  not allowed to come but they would still have been able to talk

13  and needed to talk to their relatives (.) and then there was only like 

14  15 minutes. I think it somehow caused a moral

15  conflict and distress, I’m sure. 

16 P3: Both groups of patients need their relatives but the other had- 

17  one was more privileged than the other so a sort of inequality.

18 P2: And in that kind of situation people acknowledge and

19  question why are they allowed,

19 P3: Yeah, that’s right.

One of the participants (P1) topicalises COVID-19 restrictions and claims that they were “one of the toughest things” they have faced in the ward (lines 1–2). In line 3, P2 immediately shows agreement with three confirming responses (“it has been”, “yes indeed”, “I agree”). P1 continues, stating further that what was the hardest was how the guidelines were interpreted differently. She also corrects her own talk by abruptly stopping at the word “clear” and replacing it with “or guidelines from the management”, implying that the guidelines were not, perhaps, so clear (lines 4–5). Then she questions the equality of the COVID-19 guidelines being interpreted differently by elderly care practitioners (lines 5–7). She gives a practical example of this inequality: the hospice care patients were allowed to have visitors around the clock, but palliative care patients only had 15 min of visiting time per day (lines 10–14). She further justifies her feelings by stating that clients in palliative care would still have “been able to talk and needed to talk with their relatives” (lines 12–13). She concludes that this inequality caused “a moral conflict and distress” (lines 14–15). In line 16, another participant (P3) takes a turn and presents a slightly diverging viewpoint that highlights the need for both groups of clients to be with their relatives. At the same time, she agrees with P1′s view that, in this case, the other group was more privileged, which caused the inequality (lines 16–17). Then, P2 takes a turn, which she constructs as a straight continuation of P3′s turn (note the turn-initial connector “and” in line 18), and states that such inequalities are, indeed, something that “people acknowledge and question”. These types of extensions that grammatically complete the previous sentence have been shown to display strong mutual engagement and shared understanding of the matter at hand [65].

Thus, the workshop participants considered equal treatment of clients a normative idea. The situations in which elderly care practitioners were forced to violate this ideal, such as the COVID-19 restrictions, caused MD. In daily work, the practitioners solved the dilemmatic situations differently, which caused even more contradictions. In the workshop discussions, this topic mobilised normative accounts and moral pondering among the elderly care practitioners.

When the elderly care practitioners talked about equal treatment of everyone at their workplace, the experienced inequalities engendered affective talk. Extract 4 shows an example. Before the extract, the participants had talked about the importance of positive feedback. One participant (P1) described an event in which their supervisor proposed a monthly reward system in which one of the practitioners would have been rewarded for “doing a great job”. The practitioners objected to the system because they felt that it increased inequalities among them. Two months later, the supervisor gave four nurses a present in a team-meeting to reward them for good job performance. This came as a surprise to all the practitioners, and no one knew why these four persons were considered to have performed especially well. In the first lines of the extract, P1 elaborates on her feelings related to this event.

Extract 4.

01 P1: I’m not begging to hear that I’m a good nurse, I really don’t need to,

02  but it really upset me that on what grounds did they receive that

03  reward, why? (0.2) Many others of us do

04  those same tasks in the same way but we don’t have enough time to

05  sit at a computer for two hours but apparently others have time (.)

06  that discriminates between us, unfortunately.

07 P2: Yes, but you don’t take those two hours because you don’t

08  want to dump your workload on someone else’s shoulders 

09  like they do.

10 P1: Mm, I don’t do that.

11 P3: Where do they get [that time?

12 P2: [BECAUSE THEY TAKE THAT TIME, they take it off the

13  others’ necks. The rest of us do the job and they just announce 

14  I’ll be on the computer for two hours, do not disturb.

15 P3: That’s also related to the ethical culture that those wo[rk (-_-)

16 P1: [THEY 

17  HAVE A RIGHT to act like this, there are certain things that they can 

18  just quite coldly here and they are given that opportunity.

19 P4: Others know how to take liberti[es,

20 P2: [They MOST CERTAINLY do.

P1 states that the reason for her complaint is not her desire to get compliments for “being a good nurse” but the unequal treatment of the team members. She also describes that she “felt really bad” as she did not even know why the other practitioners were rewarded, especially because the rest of the practitioners “do those same tasks in the same way” (lines 2–4). Then she moves on to complain about the team members: some people have time to “sit at a computer for two hours,” but others do not (lines 4–5). This complaint immediately invokes agreement and affiliation among the other participants. In line 7, P2 responds with the Finnish particle “nii” (translated as “yes”), claiming agreement with the position presented by P1 [66]. She affiliates with P1 by stating that she would not behave in a morally questionable way. Then she adds another element to P1′s complaint: it is a specific person among the team who internally “dumps her own workload onto others’ shoulders” (lines 8–9). P1 further positions herself as a moral person and agrees that she would not act like that (line 10). Next, P3 joins the discussion and wonders how some people have more time than others (line 11), but her turn is interrupted by P2, who states that these people “take it from the others’ necks” (lines 12–14). Like the example in Extract 1, she makes an in-situ display of anger and indignation by using a high, loud and tense voice [61]. P3 tries to return the discussion to the “ethical culture” (line 15), but her turn is interrupted again by P1 who claims, in a high, loud and tense voice, that some people are “given that opportunity” to act in this way (lines 16–18). Thus, she claims that the problem is not only those who act in a morally questionable way, but also those who permit it. P4 returns the complaint to the persons who act wrongly and “know how to take liberties” (line 19) and P2 strongly agrees (line 20).

Thus, the data example shows how the experiences of equal treatment of people at work mobilised a mutually shared affective complaint. At the same time, however, through their complaint, they invoked a strong opposition between “them” and “us”, thus contributing to the difficult relations at the workplace.

### 3.3. Collaboration

Difficulties in collaborative relationships was indeed a theme that the workshop participants explicitly topicalised. They talked about trust, respectful behaviour and helping other people. In the context of client work, the participants experienced distressing situations in which their views of good care differed from those of clients. As in Extracts 1 and 3, this topic mobilised negotiations of rights and wrongs in care work. This is exemplified by Extract 5, which starts with one of the workshop participants raising the issue of “doing too much” to help a client to eat in a situation in which eating is no longer helpful.

Extract 5.

01 P1: It still happens to some extent that we do too much in a way, 

02  that if a patient isn’t able to eat or drink, and you just try to

03  explain that they don’t have an appetite or 

04  feel thirst anymore and this is how we can still care [for them and

05 P2: [Absolutely.

06 P1: That the drip doesn’t substitute nutrition and sometimes you 

07  even need to intervene so that the relative doesn’t force-feed

08  the patient. So if [the food] just won’t go down, then that’s just how

09  it is. This still happens to some extent in my work.

10  (1.0) 

11 P1: So for example [the patient] who passed away last week 

12  that the food wouldn’t go down but they had to eat eat eat,

13 P4: You mean the patient, right?

14 P1: Yes that they consider eating so important as if it would somehow,

15 P4: Make them better, yes.

In lines 1–2, one of the participants (P1) refers to a prior conversation about the changes in work practices. She topicalises the problem of “doing too much” and that it “still happens to some extent”, implying this to be a passing phenomenon that should not exist in current healthcare. She specifies the problem as the difficulty in explaining to a relative how they can care for a client who can no longer eat or drink (lines 2–4). P2 understands the challenge (line 5), and P1 continues by demonstrating the difficulty in explaining the situation to a client. She also upgrades the problem-relevance of the topic by stating that she even needs to intervene when relatives force the clients to eat (lines 6–8). Then she returns to her initial evaluation and repeats that this happens “to some extent” in her work (line 9). When no one takes a turn (line 10), she continues by giving an example of a client who had passed away the week before and had wanted to keep eating to the very end (lines 11–12). P4 clarifies it was the client who experienced eating as important and P1 confirms, formulating the gist of her talk again [67] with “considers eating so important” (line 14). At this point (line 15), P4 collaboratively completes P1′s sentence, implying agreement between participants [64]. Thus, the diverging views about what constitutes help that supports the clients’ wellbeing invoked normative accounts and moral pondering among the elderly care practitioners. In the practitioners’ shared view, the care that the clients and relatives wished for did not always match the established care practices in certain medical situations. This engendered difficulties in communicating their views to clients and relatives.

The participants also discussed collaboration in the workplace. One reoccurring topic was the help that was given and received from colleagues. Again, this talk invoked affective expressions. Extract 6 provided a case in point. Before the extract, the participants had been pondering whether a person should go to help a teammate when they have finished their own “list of clients”. P1, who is relatively new at the workplace, tells the others about an instance in which she asked her colleagues if they tend to help others. In the first lines, she describes her reaction to their response.

Extract 6.

01 P1: I was told that it depends on who it is and was like 

02  completely shocked as I’ve never discriminated against anybody 

03  so I don’t uhm- (0.5) I think that’s really distressing.

04 P2: These are the conflicts that arise

05  during the workday and others experience it differently 

06  and agree that you should help and others say they don’t.

07  So it feels really bad.

08 P1: And I really do understand that if you really are 

09  rea::lly slow and you do it on purpose, because several people do so, 

10  and you know that there are in [the group, 

11 P3: [↑THEN YOU have to do

12  two jobs.

13 P1: Yeah, I do and I’m not going to start doing that either

14  as it’s physically and emotionally exhausting.

In lines 1–2, P1 describes how she felt in a past situation “I was completely shocked”, stating that she felt that it was discrimination and would never have done this herself. In this way, she describes the teammate’s behaviour as morally questionable. She also uses extreme-case formulations (“completely shocked”, “I’ve never”, “anybody”), which is a way of legitimising claims in interaction [68]. After a short break, she further describes her feelings, evaluating such behaviour in the workplace as "distressing” (line 3). As in Extract 1, another participant (P2) formulates the situation as a “conflict” because the team-members’ views differ (lines 4–6). Contrary to Extract 1, however, she also takes an emotional stance towards the issue and “feel(s) really bad” (line 7) affiliating with P1′s emotional stance [69]. At this point, P1 takes a diverging perspective and claims to understand that teammates refuse to help in cases in which a co-worker is “rea::lly slow” on purpose (lines 8–10). She also claims to know that “several people do so”. At this point, a third participant (P3) argues, overlapping with P1′s talk, that teammates who agree to help are forced to do the work of two people (lines 11–12). As happened in Extract 1, she makes an in-situ display of indignation by using a high, loud and tense voice [61]. P1 agrees with P3′s claim and states that she refuses to do so as it (helping others after finishing one’s own tasks) would be “physically and emotionally exhausting’ for her (lines 13–14). Thus, the workshop participant who initiated the talk by describing her shock and feeling stressed when a teammate refused to help, ends up declaring that she will refuse to help others to avoid strain.

In summary, difficulties in collaborative relations were perceived as straining. In the context of collaboration with clients, the diverging views regarding what constitutes help that supports the clients’ wellbeing invoked normative accounts and moral pondering. In the context of teammate collaboration, the diverging views regarding whether one should help one’s teammate or not mobilised emotional expressions and affective complaints.

## 4. Discussion

In this paper, we have examined the ways in which elderly care practitioners in development workshops perceived MD in their work. In terms of content, we found that the experiences of MD were related to three topics: power to influence and make decisions, equal treatment of everyone, and collaboration practices, which all occurred when talking about both client work and teamwork contexts. Our findings highlight the importance of teamwork contexts in the elderly care practitioners’ perceptions of MD. In our data, most of the discussion concerned moral dilemmas related to the teamwork context, especially elderly care practitioners’ own opportunities to influence their work, and the collaboration practices with their supervisors and colleagues. This finding aligns with those of prior research, which have linked MD among healthcare professionals to a low degree of employee autonomy [11,17]. Problems related to collaboration practices, such as lack of support from co-workers and supervisors [16] and interpersonal conflicts [70,71] seem to contribute to MD experiences. This suggests that interventions to reduce MD should not rely on one individual practitioner but on the whole work community and should involve both employees and supervisors (see also [72]). Leadership styles that emphasise care work errors as learning opportunities rather than opportunities for criticism are more likely to develop trust and commitment among care workers [18] and to ultimately lead to better communication [19] and reduced MD.

In terms of interaction, our study shows that MD experiences invoked expressions of negative emotions, including frustration and anger, which were strongly affiliated with other participants, see also [14,16,28]. The novel contribution of our research is that the negative emotional expressions were systematically related to the teamwork context. In comparison, the discussions on client work were characterised by negotiations of the rights and wrongs of care work. Understanding the emotional expressions related to MD are important, as affective outbursts may be a sign of occupational burnout [29,30]. Supporting the occupational wellbeing of elderly care practitioners is crucial, as populations in Western countries age, needs for care increase, and the number of nursing professionals decrease (e.g., [7]). Even today, elderly care practitioners experience prolonged moral distress more often than employees working in other healthcare sectors [5].

Based on our research, we suggest two solutions to reduce MD among elderly care practitioners. First, we argue that wider discussions on moral dilemmas at work are the key to reducing MD. Discussions with teammates help increase professional confidence and prepare people for the challenging situations that others have experienced [18]. In addition, these discussions are necessary to share the emotional load. As sharing negative emotional experiences at the workplace may be sensitive and challenging, work communities require psychological safety. This means a shared belief among employees that interpersonal risk-taking, such as sharing the negative experience within the team, is safe [73]. Prior research has noted that psychological safety promotes performance gains, increased learning, engagement and information-sharing, and improves satisfaction and commitment to work [66].

Second, we stress that it is essential to not only provide arenas for releasing emotions through discussions, but also to reflect on what could be improved in the organisation, and thus ascend from individual problems to institutional solutions [56]. Our data were collected in developmental workshops, the purpose of which was to identify problems in care workers’ wellbeing related to ethical work culture. The workshops aimed to develop experiments in and for the work community to solve the problems that the participants considered central in their own work. The starting points for learning were thus the theoretical premises that highlighted contradictions and triggered organisational learning [55], emphasised reflection as a significant part of learning [74] and considered employees themselves to be the initiators of solutions to the working conditions they experienced [75]. The analysis indicated that the care workers were able to reflect on their MD in a psychologically safe atmosphere, and that emotional outbursts helped them identify relevant questions and the contradictions causing MD at the teamwork level. In the future, research is needed on employee-initiated solutions to solve MD at work, and on experiences of work development for reducing MD.

### 4.1. Limitations of the Research

One strength of this study is its detailed qualitative analysis, which focuses not only on the topics of talk but also on the interactional dynamics in naturally occurring workshop discussions, but it also has certain limitations. One obvious limitation is the relatively small number of elderly care units, workshop discussions and participants in our data, which constrains the generalisability of our results. The data came from two workshop processes in one hospital district. Later, 14 similar processes were conducted in other hospital districts in Finland, and their preliminary analyses showed similar patterns of interaction. Another limitation is the lack of the participants’ demographic factors, which may have affected the ways in which they talked in the workshops (i.e., work experience). One may also ask how the specific service unit (e.g., service housing, geriatric ward), the individual work unit or the individual participants influenced the interactional patterns, especially the occurrence of strong emotional expressions. Naturally, it is possible that the cases had issues that we could not discern from the audio-recording and that involved strong outbursts of emotions. However, after going through our entire data from specifically this viewpoint, we could make no such conclusion: the emotional expressions were not restricted to specific units or individuals. Thus, possible differences between service units’ and individual practitioners’ perceptions of MD remain topics for future research.

### 4.2. Practical Implications

As our analysis shows, the source of the emotional outbursts were not the distressing situations in client work but challenges on the workplace level. Hence, attempts to improve the work-related health and wellbeing of elderly care practitioners require time and space for sharing the emotional load, followed by reflection on what could be improved in the work and what institutional solutions could help in morally distressing situations. Care workers need to be given opportunities to influence their work themselves and to find solutions that benefit their wellbeing in their everyday work. It is essential not only to provide arenas for releasing emotions and focusing on problems, but to reflect on what could be improved in the organisation, and thus create solutions to work-related wellbeing,

In the development of work, emotional outbursts should not be overly avoided, as they may provide insights into relevant issues and the contradictions behind MD at the work-community level. This creates a solid foundation for planning development experiments in work communities to reduce prolonged MD caused by elderly care practitioners’ work.

## 5. Conclusions

In terms of content, most of the MD experiences were related to teamwork, especially the elderly care practitioners’ own opportunities to influence their work and collaboration with their supervisors and colleagues. It may well be that it is easier and more normatively acceptable to share thoughts and discuss the morally dilemmatic situations concerning clients and their relatives. Thus, dilemmatic situations that concern the work and collaborative relations at the workplace may not be unburdened in everyday encounters. In addition to improving equality among elderly care practitioners and their power to influence their work and collaboration practices, more time and space are needed at work to share the emotional load. Sharing the load may relieve stress in its own right but combined with a developmental orientation that experiments with solutions to everyday work problems, it may provide ways to gain influence over one’s own work and ascend from individual problems to institutional solutions.

## Figures and Tables

**Table 1:** Number of interactional segments in three topical categories in the context of client work and teamwork.

Context	Power to Influence	Equality	Collaboration	Total
Client work	7	10	22	39
Teamwork	34	14	38	86
Total	41	24	60	125

## Data Availability

The data presented in this study are not publicly available due to privacy and ethical restrictions. Access to the data can be requested by contacting the PI of the project (J.L.).

## Appendix A

Simplified transcription symbols	
[ ]	overlapping talk
(1.0)	silence measured in seconds and tenths of a second
WORD	talk louder volume than the surrounding talk
-	abrupt cut-off of the preceding sound
@word@	spoken in an animated voice
↑↓	rise or fall in pitch
